# An allosteric pocket for inhibition of bacterial Enzyme I identified by NMR-based fragment screening

**DOI:** 10.1016/j.yjsbx.2020.100034

**Published:** 2020-07-21

**Authors:** Trang T. Nguyen, Vincenzo Venditti

**Affiliations:** a Department of Chemistry, Iowa State University, Ames, IA 50011, USA; b Roy J. Carver Department of Biochemistry, Biophysics and Molecular Biology, Iowa State University, Ames, IA 50011, USA

**Keywords:** Mixed inhibition, Competitive inhibition, Antimicrobial compounds, Principal component analysis, Bacterial phosphotransferase system, TIM barrel enzyme

## Abstract

Enzyme I (EI), which is the key enzyme to activate the bacterial phosphotransferase system, plays an important role in the regulation of several metabolic pathways and controls the biology of bacterial cells at multiple levels. The conservation and ubiquity of EI among different types of bacteria makes the enzyme a potential target for antimicrobial research. Here, we use NMR-based fragment screening to identify novel inhibitors of EI. We identify three molecular fragments that allosterically inhibit the phosphoryl transfer reaction catalyzed by EI by interacting with the enzyme at a surface pocket located more than 10 Å away from the substrate binding site. Interestingly, although the three molecules share the same binding pocket, we observe that two of the discovered EI ligands act as competitive inhibitors while the third ligand acts as a mixed inhibitor. Characterization of the EI-inhibitor complexes by NMR and Molecular Dynamics simulations reveals key interactions that perturb the fold of the active site and provides structural foundation for the different inhibitory activity of the identified molecular fragments. In particular, we show that contacts between the inhibitor and the side-chain of V292 are crucial to destabilize binding of the substrate to EI. In contrast, mixed inhibition is caused by additional contacts between the inhibitor and ⍺-helix 2 that perturb the active site structure and turnover in an allosteric manner. We expect our results to provide the basis for the development of second generation allosteric inhibitors of increased potency and to suggest novel molecular strategies to combat drug-resistant infections.

## Highlights

• EI is a central regulator of bacterial metabolism and a promising drug target.
• EI is a model system to investigate allostery in multidomain, oligomeric proteins.
• We characterize a novel allosteric pocket for inhibition of EI.
• We identify three novel allosteric inhibitors of EI.
• Inhibitor conformational flexibility determines its mechanism of action.

## Graphical abstract

**Figure:**
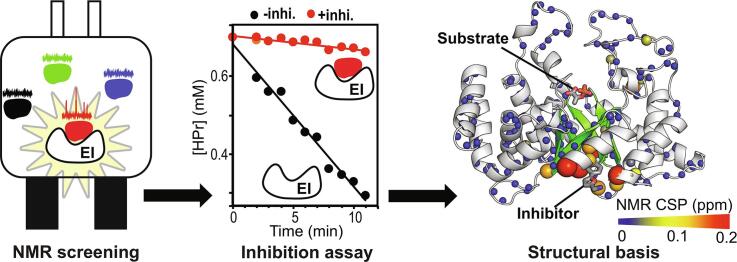

## 1 Introduction

The bacterial phosphoenolpyruvate (PEP):carbohydrate phosphotransferase system (PTS) is a signal transduction pathway that is involved in both transport and phosphorylation of a large number of carbohydrates (PTS carbohydrates), in the movement of cells toward these carbon sources (chemotaxis), in biofilm formation, in the regulation of interactions between carbon and nitrogen metabolisms, and in the regulation of a number of other metabolic pathways, including catabolic gene expression, potassium transport, and inducer exclusion (Deutscher et al., 2014, Postma et al., 1996). For all these different regulatory processes, the signal is provided by the phosphorylation state of the PTS components (Deutscher et al., 2014), which varies according to the intracellular availability of PEP (Hogema et al., 1998). PEP acts as phosphoryl donor for Enzyme I (EI), which, together with the phosphocarrier protein HPr and one of sugar-specific EIIA and EIIB pairs, forms a phosphorylation cascade that allows phosphorylation of the PTS carbohydrate bound to the membrane-spanning EIIC (Clore and Venditti, 2013). PTS-mediated regulatory mechanisms are based either on direct phosphorylation of the target protein by one of the PTS components or on phosphorylation-dependent interactions (Deutscher et al., 2014). As such, the regulatory functions of PTS are strongly impaired by inhibition of EI phosphorylation by PEP. Indeed, an *Escherichia coli* strain engineered to not express EI only grows in complex media containing cyclic adenosine monophosphate (which is needed to activate catabolic gene expression in EI-deficient strains) (Postma et al., 1996), and the growth of wild-type *E. coli*, *Pseudomonas aeruginosa*, and *Staphylococcus aureus* on Luria-Bertani (LB) or Tryptic Soy broth is severely affected by addition of EI inhibitors designed in silico (Huang et al., 2013). Moreover, a virulence study in a murine model has shown that EI-deficient strains of *Salmonella typhimurium*, *S. aureus*, and *Haemophilus influenzae* are 10 to 1000 times less virulent than wild type bacteria (Kok et al., 2003), and PTS genes have been identified on several occasions in experimental screens for virulence factors (Edelstein et al., 1999, Hava and Camilli, 2002, Jones et al., 2000, Lau et al., 2001). Therefore, potent inhibitors of EI could show antimicrobial activity by attenuating both growth rate and virulence of the infective agent. Interestingly, EI is ubiquitous and one of the best-conserved proteins in both Gram-positive and Gram-negative bacteria, and does not share any significant sequence similarity with eukaryotic proteins, making EI a possible target for development of wide-spectrum antimicrobials.

The functional form of EI is a 128 kDa symmetric dimer of identical subunits. Each subunit is composed of two structurally and functionally distinct domains separated by a long helical linker (Chauvin et al., 1996). The N-terminal domain (EIN, residues 1–249) contains the phosphorylation site (H189) and the binding site for HPr (the second PTS protein). The C-terminal domain (EIC, residues 261–575) is responsible for EI dimerization and contains the binding site for PEP. Functional regulation of EI is achieved through synergistic coupling of multiple intra and interdomain conformational equilibria that are modulated by substrate and cofactor binding. Specifically, EI undergoes (i) a monomer–dimer equilibrium (Nguyen et al., 2018, Patel et al., 2006), (ii) a compact-to-expanded equilibrium within the EIC domain (Venditti and Clore, 2012, Venditti et al., 2015b), (iii) a *g^+^*-to-*g^−^* equilibrium within the rotameric state of the H189 side chain (Suh et al., 2008), (iv) a state A-to-state B equilibrium within the EIN domain (Schwieters et al., 2010, Teplyakov et al., 2006), and (v) an open-to-close equilibrium describing a reorientation of EIN relative to EIC (Schwieters et al., 2010, Teplyakov et al., 2006, Venditti et al., 2015a, Venditti et al., 2015b). PEP binding to EIC stabilizes the dimer/compact/*g^−^*/state B/closed form of EI and activates the enzyme for catalysis (Nguyen et al., 2018, Venditti et al., 2015b). Therefore, in addition to its pharmacological relevance, EI is also an important model system for biophysical investigations on long-range allosteric communication in multi-domain, oligomeric proteins.

**Fig. 1:**
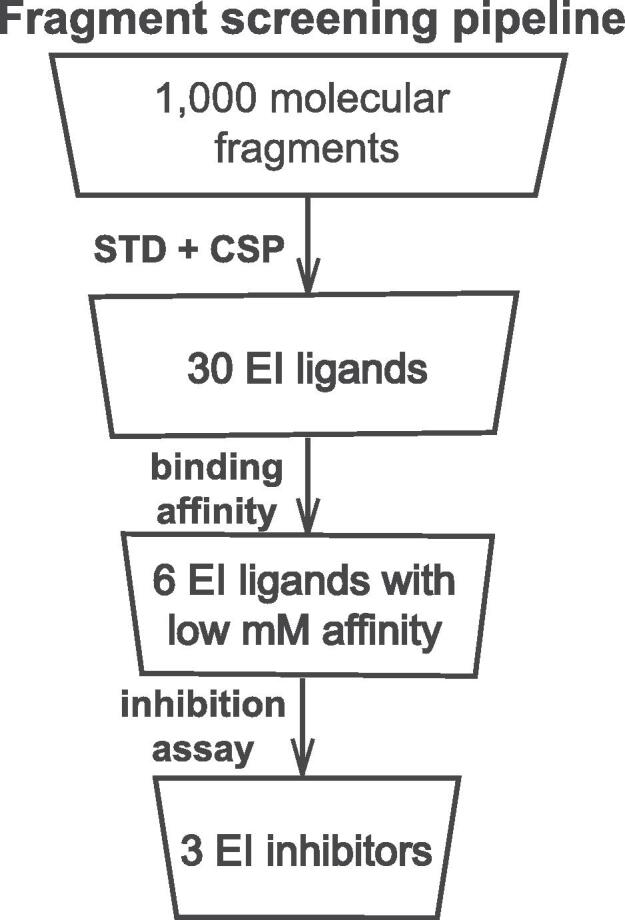
Fragment screening pipeline. Scheme of the experimental protocol employed for discovery of novel inhibitors of EI. A library of 1,000 molecular fragments is screened against EI by STD and CSP NMR experiments. The affinity of 30 positive hits for the enzyme is characterized by NMR titration experiments. 6 low-millimolar ligands are tested for their ability to inhibit the phosphoryl-transfer reaction catalyzed by the enzyme, resulting in discovery of 3 allosteric inhibitors of EI.

Here, we use NMR-based fragment screening to identify novel strategies for selective inhibition of *E. coli* EI. Starting from a library of 1000 molecular fragments, we identify three novel inhibitors of the enzyme (Fig. 1) that bind the EIC domain at a surface pocket separated more than 10 Å from the active site. Interestingly, although the three allosteric inhibitors share the same binding pocket, investigation of the reaction kinetics indicates that they inhibit the enzyme using different mechanisms. Computational studies reveal that the intrinsic flexibility of the inhibitors is chiefly responsible for their different mechanism of action, and provide hints as to how to evolve second generation inhibitors of increased potency. Such molecules will provide novel molecular tools to interrogate allosteric communication in EI and could potentially function as a new class of wide-spectrum antimicrobials.

## 2 Results and discussion

### 2.1 Identification of small-molecule ligands of EI

**Fig. 2:**
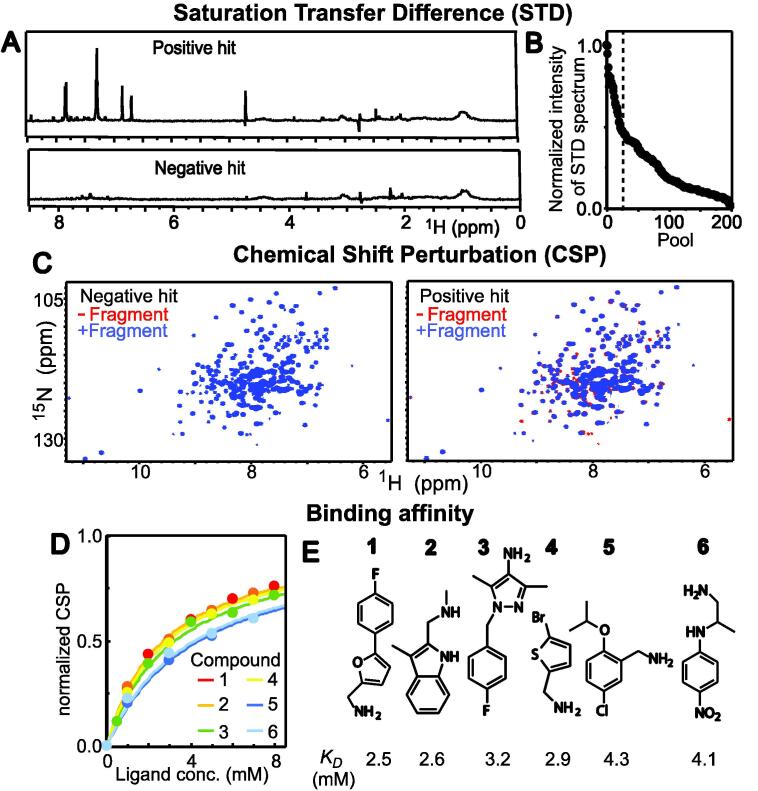
STD/CSP screening against EI. (a) Example STD-NMR spectra of a positive (top) and a negative (bottom) hit of the fragment screening. (b) The intensity of the STD spectra is plotted versus the pool index. Pools are ordered for decreasing intensity of the STD spectrum. The vertical dashed line indicates the best 25 pools that were counterscreened by CSP. STD signal intensities are normalized relative to the most intense STD spectrum. (c) ^1^H-^15^N HSQC spectra of ^15^N-labeled EIC acquired in the absence (red) and in the presence (blues) of a pool of molecular fragments. Example of a negative (left) and a positive (red) hit are provided. (d) Binding isotherms obtained for the best six ligands of EI by NMR titration experiments (compounds 1–6). Experimental data are shown as circles. Modelling of the data is shown as solid lines. Color code is red, orange, green, yellow, blue, and light blue for compound 1, 2, 3, 4, 5, and 6, respectively. (e) Line structures of compounds 1–6. *K_D_* values fitted from the binding isotherms are shown. (For interpretation of the references to color in this figure legend, the reader is referred to the web version of this article.)

Novel small-molecule ligands of EI were identified by screening a rule-of-three-compliant library of 1000 molecular fragments against EI by Saturation Transfer Difference (STD) and Chemical Shift Perturbation (CSP) NMR experiments (Carr et al., 2005). In STD NMR, the target protein is mixed with one (or more) small molecule(s) and the transfer of saturation from the protein to the small molecule is investigated by solution NMR (Mayer and Meyer, 2001). Ligand protons that are in close contact with the receptor protein receive a higher degree of saturation and generate stronger STD NMR signals. In contrast, protons that are not in contact with the target protein reveal no STD NMR signals. Therefore, STD NMR is an excellent tool to rapidly screen a small library of potential ligands against EI, as only ligands of the enzyme will return an STD NMR signal (Fig. 2a). To reduce the experimental time for STD screening, fragments were screened in pools of five, corresponding to a total of 200 NMR samples. Pools were ranked by their signal intensities, which were calculated as the sum of the intensity of the STD spectrum over the entire spectral width (Fig. 2b). The 25 pools returning the strongest STD signals were counterscreened against the enzyme by CSP experiments. These protein-detected NMR experiments are orthogonal to the ligand-detected STD experiments, and provide an independent validation for the ligand–protein interactions revealed by STD screening (Ma et al., 2016). CSP-based screening consists in measuring ^1^H-^15^N heteronuclear single quantum coherence (HSQC) spectra of the target protein in the absence and in the presence of the molecular fragment pool. Pools containing one (or more) ligand(s) of the receptor protein generate shifts of the NMR signals that are easily observable by overlaying the measured HSQC spectra (Fig. 2c) (Purslow et al., 2020). To facilitate acquisition and analysis of the NMR data, CSP experiments were measured on ^15^N-labeled samples of the isolated N- (EIN) and C-terminal (EIC) domains of EI that, being considerably smaller than the full-length protein, generate highly resolved NMR spectra characterized by high signal-to-noise ratio. In total, 30 EI ligands were identified from 20 fragment pools selected by our combined STD/CSP screening. The EI ligands were recognized from the other molecules comprising the fragment pools by comparing the pattern of the STD-NMR signals with reference ^1^H NMR spectra provided by the commercial supplier of the fragment library.

The affinity of the newly discovered EI ligands was investigated by acquiring CSP-based, NMR titration experiments on ^15^N-labeled EIN or EIC at increasing concentration of small molecule (Fielding, 2007). The data were fit using a standard equilibrium dissociation equation (Granot, 1983) to obtain the equilibrium dissociation constant (*K_D_*) per each analyzed complex (Fig. 2d). This analysis identified six small molecules that bind the EIC domain of EI with low mM affinity (Fig. 2e).

### 2.2 Identification of small-molecule inhibitors of EI

**Fig. 3:**
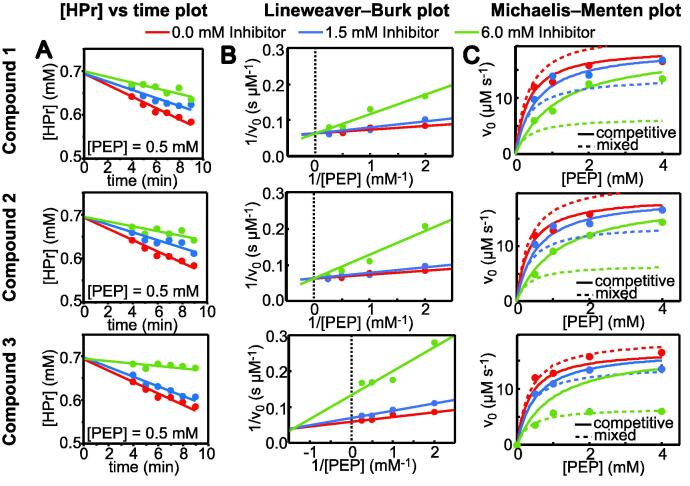
Enzyme inhibition assays. (a) Example plots showing the initial change in the concentration of unphosphorylated HPr versus time in the presence of 500 μM PEP and 0.1 μM EI. (b) Enzyme kinetic data shown as Lineweaver-Burk plots. (c) Enzyme kinetic data shown as Michaelis-Menten plots. The enzyme inhibition assays were run in the presence of compound 1 (top), 2 (center), and 3 (bottom). Data were measured at three different concentrations of inhibitor (0.0 mM, red; 1.5 mM, blues; 6.0 mM, green). Michaelis-Menten plots were modelled using a competitive (solid line) or mixed (dashed line) inhibition model (see Eqs. (3), (4)). Lineweaver-Burk plots were interpolated by linear regression, and the vertical dotted lines indicate the position of the *y*-axis. Experimental data are shown as circles. (For interpretation of the references to color in this figure legend, the reader is referred to the web version of this article.)

The six small-molecule ligands of EI identified above (compounds 1–6 in Fig. 2e) were characterized for their ability to inhibit the phosphoryl-transfer reaction from PEP to HPr catalyzed by the enzyme. The activity of full-length EI was assayed in the presence of 0.0, 1.5, and 6.0 mM of compounds 1–6 by ^1^H-^15^N SOFAST NMR experiments (Nguyen et al., 2018). This method was employed recently to study the effect of protein oligomerization on EI functional response to α-ketoglutarate binding, and allows to assay the activity of EI without interferences due to the PEP-hydrolysis reaction catalyzed by the EIC domain (Nguyen et al., 2018). Results of the enzymatic assay are reported in Fig. 3b as Lineweaver–Burk plots. In such graphs, the *y* and *x* intercepts are equivalent to the inverse of the maximum velocity (1/*V*_max_) and the negative inverse of the Michaelis constant (−1/*K_m_*), respectively. We observed that only compounds 1–3 are inhibitors of EI. In particular, compounds 1 and 2 act as competitive inhibitors (i.e. their presence increases the *K_m_* for PEP binding to EI, Fig. 3b), while compound 3 acts as mixed inhibitor of EI (i.e. its presence increases *K_m_* and decreases *V_max_* for the enzymatic reaction, Fig. 3b).

The enzyme kinetic data were modelled using a competitive inhibition model (Eq. (3)) for compounds 1 and 2, and a mixed inhibition model (Eq. (4)) for compound 3. Modelling was performed by keeping *K_m,0_* (the value of *K_m_* in the absence of inhibitor) to its literature value (350 μM) and the *K_I_*’s for compounds 1–3 to the corresponding *K_D_*’s measured by NMR titration experiments (2.5, 2.6, and 3.2 mM for compound 1, 2, and 3, respectively - Fig. 2e). *V_max,0_* (the value of *V_max_* in the absence of inhibitor) was optimized to maximize the agreement between experimental and simulated data (Fig. 3c). A best fit *V_max,0_* value of 19 ± 2 μM min^−1^ was obtained.

### 2.3 Structural basis for inhibition of EI

**Fig. 4:**
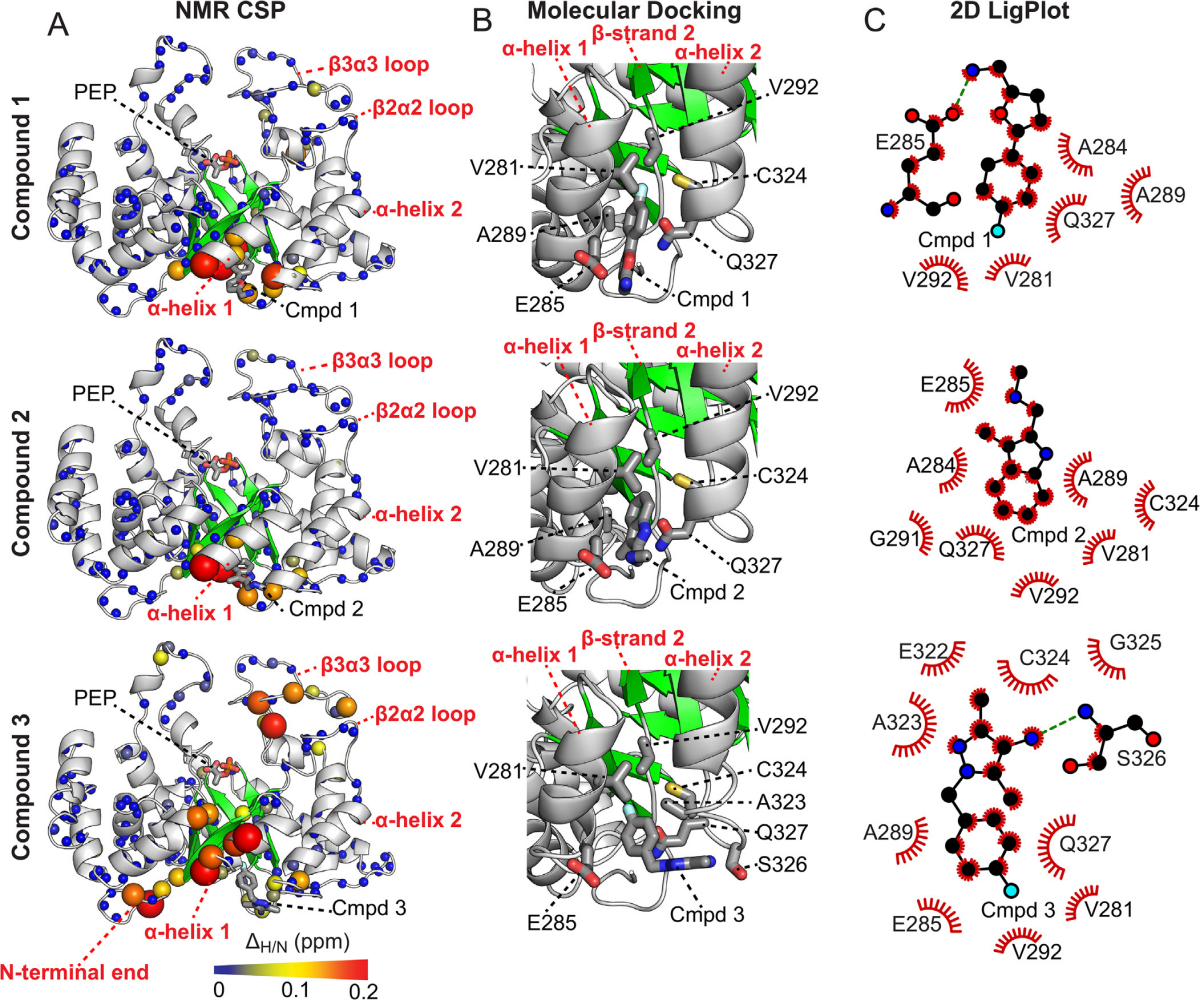
Structures of the EIC-inhibitor complexes. (a) Weighted combined chemical shift perturbations (*Δ_H/N_*) induced by 8 mM of compound 1 (top), 2 (center) and 3 (bottom) on the ^1^H-^15^N HSQC spectrum of EIC. *Δ_H/N_* values are displayed on the structure of the EIC-PEP complex as spheres with the relationship between size and color of each sphere and chemical shift perturbation depicted by the color bar. The PEP molecule is shown as solid sticks. Structure and localization of compounds 1, 2, and 3 resulting from molecular docking calculation is also displayed as solid sticks. (b) Close-up view of the inhibitor binding site. Inhibitors and EIC side-chains involved in complex formation are shown as solid sticks. (c) 2D ligand–protein interaction diagrams of the EIC-inhibitor complexes highlighting hydrophobic contacts (red) and hydrogen-bonding (dashed green line) interactions. Plots were generated using the program LigPlot (Wallace et al., 1995). (For interpretation of the references to color in this figure legend, the reader is referred to the web version of this article.)

To gain structural insight into the interaction between EI and compounds 1–3, the combined ^1^H_N_/^15^N CSP (*Δ_H/N_*) generated by 8 mM inhibitor on the ^1^H-^15^N HSQC spectrum of isolated EIC are plotted on the enzyme structure in Fig. 4. NMR chemical shifts depend on the local electronic environment at the observed nuclei. Therefore, CSP data report on local changes of the electronic structure due to the presence of the ligand or to protein conformational changes occurring upon ligand binding. Fig. 4 displays that compounds 1–3 generate large *Δ_H/N_* values at a small pocket formed by the C-terminal ends of ⍺-helix 1 (residues 268–278) and 2 (residues 310–325) of the EIC domain, suggesting that the three molecules share the same binding site. Interestingly, this surface pocket is located greater than 10 Å away from the binding site for PEP (Fig. 4), indicating that compounds 1–3 perturb the affinity of the EI-PEP complex (Fig. 3) in an allosteric manner. Of note, compound 3 generates additional CSP at the N-terminal end of the EIC domain and at the β3α3 loop of the active site (Fig. 4). This observation suggests that compound 3 induces conformational changes at the active site of EI that might be responsible for the ability of compound 3 to reduce the *V_max_* for the phosphoryl-transfer reaction (Fig. 3). Alternatively, the additional CSP induced by compound 3 might indicate the existence of a second binding site on EIC. However, we tend to exclude this hypothesis for two reasons: (i) fitting CSP titration data for the residues in the allosteric binding pocket and for the residues in the β3α3 loop separately returns *K_D_* values that are identical within experimental error (3.2 ± 0.2 mM and 3.5 ± 0.3 mM, respectively), and (ii) MD simulations data indicate that binding of compound 3 to the pocket at the C-terminal ends of ⍺-helices 1 and 2 perturbs the structure of the β3α3 loop (see below).

Atomic-resolution structural models for the complexes formed by EIC with compounds 1–3 were constructed by molecular docking of one copy of the ligand into the binding pocket defined by CSP data (i.e. the surface pocket defined by the C-terminal ends of ⍺-helix 1 and 2, Fig. 4a). Calculations were run with AutoDock as described in Methods, and the resulting structures are displayed in Fig. 4. As expected from the close similarity of the chemical structures of the three ligands (Fig. 2e), compounds 1–3 adopt a similar binding mode on EIC. Indeed, for all ligands, the hydrophobic aromatic ring dives into the hydrophobic pocket formed by ⍺-helices 1 and 2, while hydrophilic groups remain solvent exposed. Of note, all small molecules make close contacts with the side chain of V292 (Fig. 4), which is located at the N-terminal end of β-strand 2 (residues 292–296). As β-strand 2 is responsible for two key interactions that stabilize binding of PEP to EIC (namely: an hydrophobic contact between the L294 side-chain and the CH_2_ group of PEP, and a salt-bridge between the side chain of R296 and the phosphate group of PEP) (Venditti and Clore, 2012, Venditti et al., 2013), these contacts between the inhibitors and V292 can perturb the structure and or the dynamics of the PEP binding site and be responsible for the effects of compounds 1–3 on the *K_M_* of the EIC-PEP complex. Significant differences in the way compounds 1–3 bind the enzyme are observed at the level of ⍺-helix 2. Indeed, while compounds 1 and 2 make minimal contacts with the C-terminal end of ⍺-helix 2, the presence of the sp^3^ C bridging the two aromatic groups of compound 3 allows this inhibitor to bend and form extensive contacts with S326 and Q327 (Fig. 4). As this helix is directly connected to the protein active site via the β2α2 loop (which directly contacts the active site β3α3 loop), we hypothesize that the interactions established by compound 3 with S326 and Q327 perturb the structure of the active-site and are, therefore, responsible for the effect of this inhibitor on *V_max_*.

**Fig. 5:**
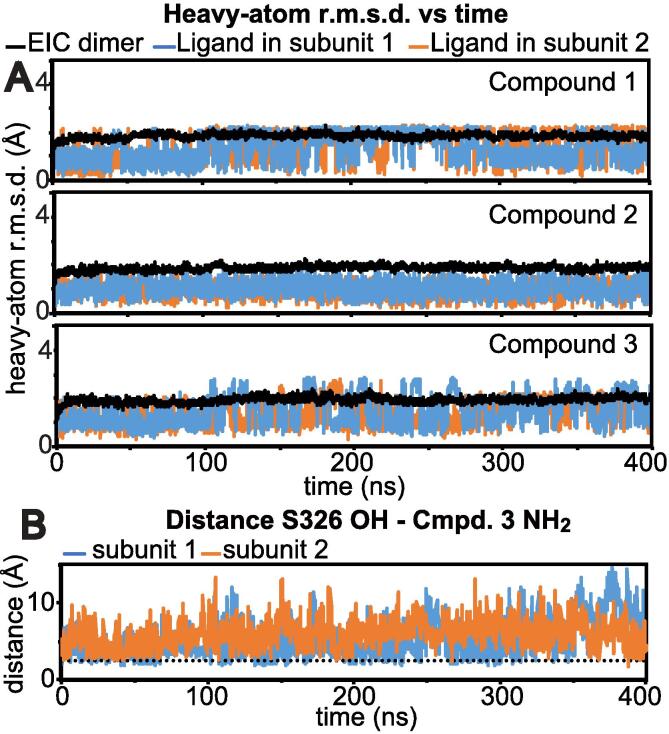
MD simulations of the EIC-inhibitor complexes. (a) Heavy-atom r.m.s.d. to the docking structure versus time calculated for the 400-ns MD run on the EIC-inhibitor complexes. EIC was simulated in its physiological dimeric form with inhibitors bound to both subunits. The r.m.s.d. calculated for the EIC, the inhibitor bound to the first subunit, and the inhibitor bound to the second subunit are colored black, light blue, and orange, respectively. Top, center, and bottom plots are for the complexes with compound 1, 2, and 3, respectively. (b) The distance between the hydroxyl group of S326 and the amine group of compound 3 is plotted versus time. Data for subunit 1 and 2 are colored light blue and orange, respectively. The dotted line is at 2.4 Å to indicate the distance required for hydrogen-bond formation. (For interpretation of the references to color in this figure legend, the reader is referred to the web version of this article.)

The structural basis for the mixed inhibition of EI caused by compound 3 has been investigated further by means of Molecular Dynamics (MD) simulations. In particular, 400-ns long MD simulations were run by using the docking EIC-inhibitor complexes as the starting structures. EIC was simulated in its physiological, dimeric form (Nguyen et al., 2018, Patel et al., 2006, Venditti and Clore, 2012) with inhibitors bound to both subunits. Stability of the simulations was evaluated by plotting the heavy-atom root mean square deviation (r.m.s.d.) from the starting structure versus time (Fig. 5a). Analysis of these plots highlights the greater rigidity of compound 2 compared to compounds 1 and 3, which show recurrent transitions to alternative rotameric structures (note the sharp transitions in r.m.s.d. versus time observed for compounds 1 and 3 in the MD simulations). Despite this intrinsic flexibility, the hydrophilic ring of compound 3 contacts the C-terminal end of ⍺-helix 2 for the entire 400-ns trajectory, as evidenced by the fact that the hydrogen-bond between the hydroxyl group of S296 and the amine group of the inhibitor persists for the majority of the MD simulation (Fig. 5b).

**Fig. 6:**
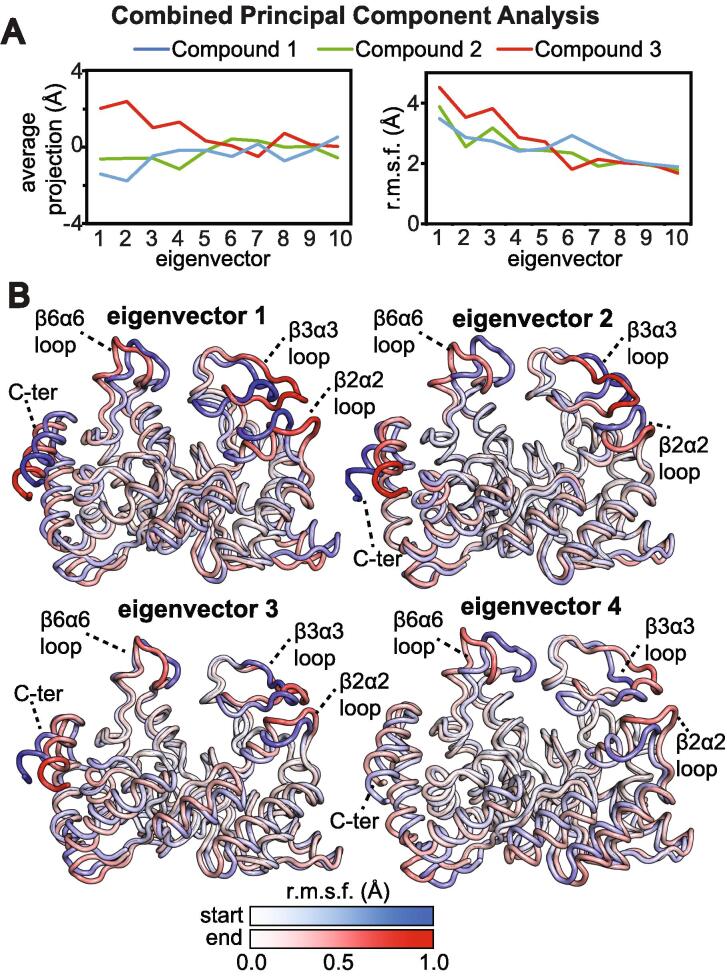
Combined PCA analysis of the MD trajectories. (a) Average projection (left) and root mean square fluctuations (r.m.s.f.) relative to the average structure (right) obtained by projecting the MD trajectories of EIC bound to compound 1 (blue), 2 (green), and 3 (red) on a common set of eigenvectors obtained from the concatenated trajectory (see main text). Combined PCA analysis was performed using the coordinates of the C_⍺_ atoms of EIC. Results for the first 10 eigenvectors are shown. (b) Start (blue) and end (red) points of the pseudo-trajectories describing eigenvectors 1–4. Residue-specific r.m.s.f. values (relative to the average structure) in the eigenvector calculated over the concatenated trajectory are plotted as color gradient on the start and end structures to emphasize the specific contribution of different EIC regions to each eigenvector. (For interpretation of the references to color in this figure legend, the reader is referred to the web version of this article.)

To analyze if EIC undergoes different dynamics when bound to the three small molecule inhibitors, we performed a ‘combined’ principal component analysis (PCA) on the simulated trajectories (van Aalten et al., 1995). In this method, two or more trajectories (fitted on the same reference structure) are concatenated, and a covariance matrix is constructed and diagonalized to obtain a common set of eigenvectors, describing the variance of the atomic coordinates in the combined MD simulation, and eigenvalues, describing the extent of the atomic fluctuations in the corresponding eigenvectors. When PCA is performed on a concatenated trajectory and eigenvectors are ordered by decreasing eigenvalue, significant differences in the structure and dynamics of the simulated systems (in our case the three EIC-inhibitor complexes) are described by the first few eigenvectors (van Aalten et al., 1995). In the particular case of EIC, we have created three 800-ns trajectories (one per each EIC-inhibitor complex) by appending the 400-ns trajectory of the second subunit to the 400-ns trajectory of the first subunit of the EIC dimer. These three 800-ns trajectories were concatenated together and investigated by combined PCA performed on the coordinates of C_α_ atoms of EIC. Once a common set of PC’s are obtained, the separate 800-ns trajectories are projected onto the resulting eigenvectors, and the properties of these projections are compared for all simulations. In particular, there are two main quantities of interest: the average projection and the root mean square fluctuation (r.m.s.f.) in the projection. Differences in the average projection on a particular eigenvector indicate that the simulations have different average displacement (i.e. average structure) in that PC. In contrast, r.m.s.f. differences in a particular eigenvector indicate that the simulations have different dynamics in the collective motion described by that PC. Analysis of the first 10 PC’s indicates that the simulated EIC-inhibitor complexes have similar molecular dynamics (i.e. similar r.m.s.f. versus eigenvector plots) but different equilibrium structures (Fig. 6a). In particular, structural changes are described by the first four PC’s, in which EIC bound to compound 3 has average projections considerably different from the ones of EIC bound to compounds 1 and 2 (Fig. 6a). The collective motions described by the first four PC’s are displayed in Fig. 6b by superimposing the start and the end frames of the pseudo-trajectory describing each eigenvector. A pseudo-trajectory with a negative average displacement has an equilibrium structure shifted toward the start point of the concerted motion, while a positive average displacement indicates that the average structure of the pseudo-trajectory is shifted toward the end point of the concerted motion. Inspection of Fig. 6 reveals that the first four PC’s describe collective motions involving the C-terminal helix and the active site β2α2, β3α3, and β6α6 loops of EIC. In particular, the active site β3α3 loop undergoes a closed-to-open (start-to-end) conformational equilibrium on PC’s 1 and 4, with compounds 1 and 2 favoring the closed conformation (note that the average projections of the simulations with compounds 1 and 2 on PC’s 1–4 are negative) and compound 3 favoring the open conformation (note that the average projections of the simulation with molecule 3 on PC’s 1–4 are positive). As the β3α3 loop of EIC has to adopt a fully closed configuration for efficient EI catalysis (Dotas et al., 2020), we ascribe the mixed inhibitor behavior of compound 3 to its ability to destabilize the catalytically-competent, fully closed conformation of the β3α3 loop.

## 3 Conclusion

EI is emerging as an important model system to study allosteric regulation in multidomain, oligomeric enzymes, and as a promising pharmaceutical target for antimicrobial design. In this contribution, we have characterized a novel surface pocket localized on the EIC domain that is allosterically coupled to the enzyme active site. By using NMR-based fragment screening, we identify three small molecules (referred to as compounds 1, 2, and 3) that bind to the allosteric pocket and inhibit the phosphoryl-transfer activity of EI. Interestingly, the *K_D_* values measured for the three EIC-inhibitor complexes (~3 mM) are comparable to the equilibrium dissociation constant reported for the EIC interaction with α-ketoglutarate (~2 mM) (Venditti et al., 2013), a metabolite that acts simultaneously as a competitive inhibitor and an allosteric stimulator of the enzyme (Nguyen et al., 2018), and that was shown to regulate the activity of EI in vivo (Doucette et al., 2011, Nguyen et al., 2018). Therefore, the inhibitors identified here can be used as chemical probes to investigate long-range communication in EI.

**Fig. 7:**
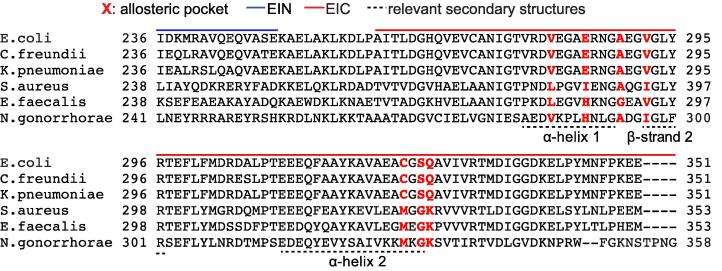
EI sequence alignment. The sequence of the *E. coli* EI is aligned against EI from other, randomly selected, drug-resistant bacteria: *Citrobacter freundii* (identity 97%; similarity 99%; gaps 0%), *Klebsiella pneumoniae* (identity 96%; Similarity 98%; gaps 0%), *Staphylococcus aureus* (identity 51%; similarity 70%; gaps 0%), *Enterococcus faecalis* (identity 49%; similarity 68%; gaps 1%), and *Neisseria gonorrhorae* (identity 36%; similarity 58%; gaps 2%). Residues forming the allosteric pocket are in red. Blue and red lines indicate the EIN and EIC domains, respectively. Dashed lines indicate the locations of ⍺-helices 1 and 2 and β-strand 1. Full sequence alignments are shown in Supplementary Fig. S1. (For interpretation of the references to color in this figure legend, the reader is referred to the web version of this article.)

On the other hand, testing the druggability of the allosteric pocket identified here for antimicrobial applications will require evolution of compounds 1–3 into second generation inhibitors of increased potency. In this respect, several hints for the development of second generation allosteric inhibitors can be inferred from the computational studies on the EIC-inhibitor complexes summarized in Results and discussion. Importantly, the presence within the inhibitor of a hydrophobic, six-membered aromatic ring and a more hydrophilic moiety seems crucial for orienting the molecule inside the aromatic pocket. In particular, it is imperative that the inhibitor penetrates deep enough into the pocket to form contacts with V292, which allosterically alter the properties of the PEP binding site and reduce the affinity of EI for its substrate. In addition, we notice that formation of contacts between the inhibitor and the C-terminal end of ⍺-helix 2 allosterically perturbs the structure of the active site at the β3⍺3 loop. As the β3⍺3 loop is directly involved in stabilization of the catalytic transition state (Dotas et al., 2020), this structural rearrangement negatively affects the efficiency of the enzyme by reducing its turnover number. Our docking and MD results suggest that introducing a flexible element between the hydrophobic and hydrophilic moieties of the inhibitor (such as, for example, the sp^3^ C in compound 3) favors formation of extensive contacts with ⍺-helix 2 by allowing the small molecule to adopt a bent conformation. In alternative, branched molecules could be designed starting from compounds 1 and 2 to increase their interactions with the C-terminal end of ⍺-helix 2 and confer mixed inhibitor character to these second generation compounds. Finally, in silico screening campaigns targeting the allosteric site at the C-terminal end of ⍺-helixes 1 and 2 of EIC might provide additional clues toward evolution of compounds 1–3 and/or suggest novel lead compounds for inhibition of EI. As the allosteric pocket identified here is conserved across EI from several bacterial strains (including important drug-resistant organisms) (Fig. 7), the results and strategies presented in this work may inspire new, much needed, molecular routes to inhibition of bacterial infections. However, as the allosteric pocket is not fully conserved in all bacterial species, further investigations are required to investigate the effect of single-point mutations within the allosteric pocket on the activity and inhibition of EI. These studies will inform on the ability of the allosteric inhibitors developed here to inhibit EI from different bacterial strains and to withstand drug resistance mechanisms.

## 4 Methods

### 4.1 Protein expression and purification

*E. coli* EI and uniformly ^15^N-labelled EIC, EIN, and HPr were expressed and purified as previously described (Nguyen et al., 2018, Suh et al., 2008, Venditti and Clore, 2012).

### 4.2 Fragment preparation

A commercial library of 1000 molecular fragments was purchased from ChemBridge. The library was designed to meet the “rule of three” for fragment-based screening (i.e., molecular weight less than 300 Da, number of hydrogen bond donors and acceptors does not exceed 3, and cLogP value less than 3) (Jhoti et al., 2013). To speed up the screening procedure, the 1000 fragments were pooled in groups of 5. The composition of each pool was optimized to reduce the risk of overlap among the ^1^H NMR signals of the molecular fragments. Stock solutions were prepared by dissolving each pool in DMSO‑d_6_ so that each fragment is at final concentration of 2.5 mM.

### 4.3 NMR spectroscopy

All spectra were acquired on Bruker 800 MHz spectrometers equipped with a z-shielded gradient triple resonance cryoprobe. ^1^H-^15^N HSQC spectra of free EIN, EIC, and HPr were assigned according to previously reported NMR chemical shifts (Garrett et al., 1999, Venditti and Clore, 2012, Venditti et al., 2011). NMR samples for STD screening were prepared in 20 mM phosphate buffer (pD 7.8), 100 mM NaCl, 4 mM MgCl_2_, and 99.9% D_2_O. The protein concentration was 10 µM. A total of 200 NMR samples were prepared by adding 40 µL of fragment pool stock solution (prepared as described above) directly into the 500 µL (final volume) NMR sample (note that the final concentration of each fragment in the NMR sample was 200 µM). The ^1^H STD spectra were measured at 37 °C by applying a selective saturation field for 400 ms at 20 and 0.9 ppm for the off-resonance and the on-resonance experiment, respectively. Acquisition was automated by using an autosampler. Spectra were processed and analyzed by using MestReNova 14 (https://mestrelab.com/software/mnova/).

CSP screening experiments were run at 37 °C in 20 mM Tris (pH 7.4), 100 mM NaCl, 4 mM MgCl_2_, 2 mM DTT, and 5% D_2_O. The protein concentration was 400 µM. A total of 40 µL of fragment pool stock solution were added to the 500 µL NMR sample.

NMR titration experiments were measured at 37 °C in 20 mM Tris (pH 7.4), 100 mM NaCl, 4 mM MgCl_2_, 2 mM DTT, and 5% D_2_O. The protein concentration was 400 µM and the concentration of small molecule was varied between 0 and 8 mM. Spectra were processed using NMRPipe (Delaglio et al., 1995) and analyzed using the program SPARKY (http://www.cgl.ucsf.edu/home/sparky). Assignment of the ^1^H-^15^N cross-peaks for the EIC-small molecule complexes was performed by titration experiments, following the change in ^1^H-^15^N cross-peak positions as a function of added small molecule. Weighted combined ^1^H/^15^N chemical shift perturbations (*Δ_H/N_*) resulting from the addition of increasing concentrations of small molecule were calculated using the following equation (Mulder et al., 1999):

(1) $$\Delta_{H/N}=\sqrt{{\left({\Delta \delta }_{H}W_{H}\right)}^{2}+{\left({\Delta \delta }_{N}W_{N}\right)}^{2}}$$

where *W_H_* (=1) and *W_N_* (=0.154) are weighting factor for the ^1^H and ^15^N amide shifts, respectively. *Δδ_H_* and *Δδ_N_* are the ^1^H and ^15^N chemical shift differences in ppm, respectively, between free and bound states. The equilibrium dissociation constants (*K_D_*) for the EIC-inhibitor complexes were obtained by fitting the changes in *Δ_H/N_* with increasing concentration of small molecule using the equation (Granot, 1983):

(2) $$\Delta_{H/N}=\Delta_{0}\frac{P+L+K_{D}-\sqrt{{\left(P+L-K_{D}\right)}^{2}-4PL}}{2P}$$

where Δ*_0_* is the weighted combined ^1^H/^15^N chemical shift at saturation, and *P* and *L* are the protein and small molecule concentrations, respectively.

### 4.4 Enzymatic assay

Enzyme kinetic assay we run by measuring the rate of phosphoryl transfer from PEP to HPr catalyzed by full-length EI by using ^1^H-^15^N SOFAST NMR spectra as described previously (Nguyen et al., 2018). Reaction were run at 25 °C in 20 mM Tris (pH 7.4), 100 mM NaCl, 4 mM MgCl_2_, 2 mM DTT, and 5% D_2_O. The assay was performed in triplicate. The enzyme kinetic data measured at different concentration of inhibitor were fit using a completive or mixed inhibition model:

(3) $$Competitive\;inhibition-v_{0}=\frac{V_{\max,0}\left[PEP\right]}{K_{m,0}\left(1+\frac{\left[I\right]}{K_{I,S}}\right)+\left[PEP\right]}$$

(4) $$Mixed\;inhibition-v_{0}=\frac{V_{\max,0}\left[PEP\right]}{K_{m,0}\left(1+\frac{\left[I\right]}{K_{I,S}}\right)+\left[PEP\right]\left(1+\frac{\left[I\right]}{K_{I,I}}\right)}$$

where *v_0_* is the initial velocity of the enzymatic reaction, *[PEP]* and *[I]* are the concentration of substrate and inhibitor, respectively, *K_I,s_* is the equilibrium dissociation constant for the enzyme-inhibitor complex, *K_I,I_* is the dissociation constant for the interaction between the inhibitor and the enzyme-PEP complex, and *K_m,0_* and *V_max,0_* are the Michaelis constant and maximum velocity in the absence of inhibitors, respectively. The seven measured datasets (in the absence of inhibitor, and in the presence of two concentrations of each inhibitor) were fit globally. In the global fitting procedures *K_m,0_* was kept fixed to its experimental value (350 μM), *K_I,I_* and *K_I,S_* were kept fixed to the value of *K_D_* measured for the EI complexes with compounds 1–3 by NMR CSP experiments, and *V_max,0_* was optimized to maximize the agreement between experimental and simulated data.

### 4.5 Molecular docking simulations

Molecular docking simulations were run using the coordinates of the EIC domain from the crystallographic structure of the full-length *E. coli* EI (PDB: 2HWG) as the target. Before the actual docking run, the protein structure was energy minimized by 1000 steps of steep descended followed by 1000 steps of conjugated gradient algorithm. Energy minimization was performed using the Amber 16 simulation package (Case et al., 2005) and the Amber ff14SB force field (Maier et al., 2015). The structures of compounds 1–3 were docked into the protein using AutoDockTools 1.5.4 and Autodock 4.2 (Morris et al., 2009). A cubic grid box (grid spacing = 0.373 Å; 40 × 40 × 40 grid points) was placed at the C-terminal ends of ⍺-helix 1 and 2 of EIC. Docking was performed using the Lamarckian genetic algorithm (LGA) and allowing the side chains of E285 and N327 conformational flexibility during the simulations. For the small molecules, the 5 and 6-membered aromatic rings were considered rigid, while all other bonds were treated as rotatable. Most of docking parameters were kept as default, with the exception of the population size (set to 150 with 2,500,000 evaluations) and the maximum number of generations (set to 27,000). Cluster analysis was performed with a r.m.s.d. tolerance of 2 Å. The best conformation is considered to be the conformation with the lowest free energy of binding.

### 4.6 Molecular dynamics simulations

The structures of the EIC-inhibitor complexes obtained by molecular docking simulations were used as the starting point for 400 ns MD simulations ran using the Amber 16 package (Case et al., 2005) and the Amber ff14SB force field (Maier et al., 2015). EIC was simulated in its dimer form with inhibitors bound to both subunits. The small molecules were parameterized with the AM1-BCC charge model (Jakalian et al., 2002) and the GAFF force field (Huang and Roux, 2013). The initial complex was centered in a truncated octahedron, filled with TIP3P water model (Mark and Nilsson, 2001) and neutralizing ions, and the distance between the protein atoms and the boundaries was set to 10 Å. Energy minimization of the initial structures, including 1000 steps of steepest descent and 1000 steps of conjugate gradient, was performed in 3 stages. First, ions and water positions were relaxed. Then, the EIC-inhibitor complex was allowed to relax. Finally, the full system was energy minimized. The system was equilibrated with a 1 ns run in which the temperature was gradually raised from 0 to 310 K, followed by a 5 ns run in which the temperature was held constant at 310 K. The equilibrated system was simulated for 400 ns by keeping the temperature (310 K) and pressure (1 atm) constant. Periodic boundary conditions were applied, and bonds were restrained with the SHAKE algorithm (Ryckaert et al., 1977). An integration step of 2 fs was used. Weak coupling to an external pressure and temperature bath was used (Berendsen et al., 1984). Particle-Mesh Ewald summation with a cutoff of 10 Å for long-range interactions was used to treat electrostatic interactions (Essmann et al., 1995).

Analysis of the MD trajectories was performed in Amber 16 using the CPPTRAJ tool (Roe and Cheatham, 2013). CPPTRAJ was also employed for combined PCA analysis (van Aalten et al., 1995). Analysis was performed on the C_α_ atoms using the protocol described at https://amberhub.chpc.utah.edu/introduction-to-principal-component-analysis/.

## Declaration of Competing Interest

The authors declare that they have no known competing financial interests or personal relationships that could have appeared to influence the work reported in this paper.
