# Supplementary material for: An allosteric pocket for inhibition of bacterial Enzyme I identified by NMR-based fragment screening
Source: J Struct Biol X. 2020 Jul 21;4:100034. doi: 10.1016/j.yjsbx.2020.100034 (PMC7385036; doi:10.1016/j.yjsbx.2020.100034)
Supplement: Supplementary data 1 [file mmc1.pdf]

SUPPLEMENTARY INFORMATION

An allosteric pocket for inhibition of bacterial Enzyme I identified by NMR-based fragment screening

Trang T. Nguyen[1] and Vincenzo Venditti*[,1,2]

[1] *Department of Chemistry, Iowa State University, Ames, Iowa 50011, USA.*

[2] *Roy J. Carver Department of Biochemistry, Biophysics and Molecular Biology, Iowa State University, Ames, Iowa 50011, USA.*

[*] Address correspondence to:

Vincenzo Venditti, Department of Chemistry, Iowa State University, Hach Hall, 2438 Pammel Drive, Ames, IA 50011, USA. email: venditti@iastate.edu; Tel. 515-294-1044; Fax: 515-294-7550; ORCID 0000-0001-8734-0400.

Keywords: Mixed inhibition; competitive inhibition; antimicrobial compounds; Principal Component Analysis; bacterial phosphotransferase system; TIM barrel enzyme

```
E.coli          1   MISGILASPGIAFGKALLLKEDEIVIDRKKISADQVDQEVERFLSGRAKASAQLETIKTK  60
C.freundii      1   MISGILASPGIAFGKALLLKEDEIVIDRKKISADKVDQEVERFLSGRAKASAQLEAIKTK  60
K.pneumoniae    1   MISGILASPGIAFGKALLLKEDEIVIDRKKISADKVDQEVERFLSGRAKASAQLEVIKTK  60
S.aureus        4   LIKGIAASDGVAIAKAYLLVEPDLTFDKNEKVTD-VEGEVAKFNSAIEASKVELTKIRNN  62
E.faecalis      4   MLKGIAASDGVAVAKAYLLVQPDLSFN--KTSVEDTDAEATRLDDALAKSTEELQAIRDK  61
N.gonorrhorae   4   VLHGVAAGKGIAVGCAHLIARGTEEVPQYDVAQADTDAEAERFDAAVKATRKELEQLRSA  63

E.coli          61  AGETFGEEKEAIFEGHIMLLEDEELEQEIIALIKDKHMTADAAAHEVIEGQASALEELDD 120
C.freundii      61  AGETFGEEKEAIFEGHIMLLEDEELEQEIIALIKDKHMTADAAAHEVIEGQATALEELDD 120
K.pneumoniae    61  AGETFGEEKEAIFEGHIMLLEDEELEQEIIALIKDKHMTADAAANEVIDGQATALEELDD 120
S.aureus        63  AEVQLGADKAAIFDAHLLVLDDPELIQPIQDKIKNENANAATALTDVTTQFVTIFESMDN 122
E.faecalis      62  AAQSLGEAEAQVFDAHLMVLSDPEMVGQIKQNIQDNKVNAEAALKEVTDMYIGMFEAMDD 121
N.gonorrhorae   64  IPENAPTELGAFISLHLMLLTDVTLSREPVDILREQKINAEWALKQQSDKLAAQFDNMDD 123

E.coli          121 E-YLKERAADVRDIGKRLLRNILG----LKIIDLSAIQDEVILVAADLTPSETAQLNLKK 175
C.freundii      121 E-YLKERAADVRDIGKRLLRNILG----LAIIDLSAIQEEVILVAADLTPSETAQLNLNK 175
K.pneumoniae    121 E-YLKERAADVRDIGKRLLRNILG----LAIIDLSAIQDEVILVAADLTPSETAQLNLKK 175
S.aureus        123 E-YMKERAADIRDVSKRVLSHILG----VELPNPSMIDESVVIVGNDLTPSDTAQLNKEF 177
E.faecalis      122 NAYMQERAADIRDVAKRILAHLLG----VTLPNPSMINEEVIVVAHDLTPSDTAQLDRTY 177
N.gonorrhorae   124 A-YLRERKQDMLQVVRRIHNNLIGQGNELEVAD--NLFDETVLIANDLSPADTVLFKEQR 180

E.coli          176 VLGFITDAGGRTSHTSIMARSLELPAIVGTGSVTSQVKNDDYLILDAVNNQVYVNPTNEV 235
C.freundii      176 VLGFITDAGGRTSHTSIMARSLELPAIVGTGSVTSQVKNDDYLILDAVNNQVYVNPTNDV 235
K.pneumoniae    176 VLGFITDAGGRTSHTSIMARSLELPAIVGTGSITAQVKNGDYLILDAVNNQVLINPSNEQ 235
S.aureus        178 VLGFATNIGGRTSHSAIMSRSLEIPAIVGTKSITQEVKQGDMIIVDGLNGDVIVNPTEDE 237
E.faecalis      178 VKAFVTDIGGRTSHSAIMARSLEIPAIVGTKEITDKVKAGDILAVNGIIGDVIIDPTDAE 237
N.gonorrhorae   181 IAAFVTDAGGPTGHTAILGRSLDIPSVVGLHNARKLITEGETVIVDGINGVLIISPDESV 240

E.coli          236 IDKMRAVQEQVASEKAELAKLKDLPAITLDGHQVEVCANIGTVRDVEGAERNGAEGVGLY 295
C.freundii      236 IEQLRAVQEQVATEKAELAKLKDLPAITLDGHQVEVCANIGTVRDVEGAERNGAEGVGLY 295
K.pneumoniae    236 IEALRSLQAQVAEEKAELAKLKDLPAITLDGHQVEVCANIGTVRDVEGAERNGAEGVGLY 295
S.aureus        238 LIAYQDKRERYFADKKELQKLRDADTVTVDGVHAELAANIGTPNDLPGVIENGAQGIGLY 397
E.faecalis      238 KSEFEAEAKAYADQKAEWDKLKNAETVTADGKHVELAANIGTPKDLEGVHKNGGEAVGLY 297
N.gonorrhorae   241 LNEYRRRAREYRSHKRDLNKLKKTAAATADGVCIELVGNIESAEDVKPLHNLGADGIGLF 300

E.coli          296 RTEFLFMDRDALPTEEEQFAAYKAVAEACGSQAVIVRTMDIGGDKELPYMNFPKEE---- 351
C.freundii      296 RTEFLFMDRESLPTEEEQFAAYKAVAEACGSQAVIVRTMDIGGDKELPYMNFPKEE---- 351
K.pneumoniae    296 RTEFLFMDRDALPTEEEQFAAYKAVAEACGSQAVIVRTMDIGGDKELPYMNFPKEE---- 351
S.aureus        298 RTEFLYMGRDQMPTEEEQFEAYKEVLEAMGGKRVVVRTLDIGGDKELSYLNLPEEM---- 353
E.faecalis      298 RTEFLYMDSSDFPTEEDQYQAYKAVLEGMEGKPVVVRTMDIGGDKELPYLTLPHEM---- 353
N.gonorrhorae   301 RSEFLYLNRDTMPSEDEQYEVYSAIVKKMKGKSVTIRTVDLGVDKNPRW--FGKNSTPNG 358

E.coli          352 --NPFLGWRAIRIAMDR--REILRDQLRAILRASAFGKLRIMFPMIISVEEVRALRKEIE 407
C.freundii      352 --NPFLGWRAVRIAMDR--KEILRDQVRAILRASAFGKLRIMFPMIISVEEVRALRKEIE 407
K.pneumoniae    352 --NPFLGWRAVRIAMDR--KEILRDQVRAILRASAFGKLRIMFPMIISVEEVRALKKEIE 407
S.aureus        354 --NPFLGWRAIRLCLAQ--QDIFRPQLRALLRASVYGKLNIMFPMVATINEFREAKAILL 409
E.faecalis      354 --NPFLGWRALRISLSELGDGMFRTQMRALLRASVHGNLRIMFPMVATLKEFRAAKAIFE 411
N.gonorrhorae   359 SLNPALGMTGIRLCLAE--PVMFRTQMRAILRAAVHGPVRMMWPMITSVSEVRQCLIHLD 415

E.coli          408 IYKQELRDEGKAFDESIEIGVMVETPAAATIARHLAKEVDFFSIGTNDLTQYTLAVDRGN 467
C.freundii      408 IYKQELRDEGKAFDESIEIGVMVETPAAATIARHLAKEVDFFSIGTNDLTQYTLAVDRGN 467
K.pneumoniae    408 IYKQELRDEGKAFDESIEIGVMVETPAAATIARHLAKEVDFFSIGTNDLTQYTLAVDRGN 467
S.aureus        410 EEKENLKNEGHDISDDIELGIMVEIPATAALADVFAKEVDFFSIGTNDLIQYTLAADRMS 469
E.faecalis      412 DEKQKLVNEGVEVSNDIQVGIMIEIPAAAVLADKFAKEVDFFSVGTNDLIQYTMAADRMN 471
N.gonorrhorae   416 TAQRQLAERGDAFGE-VGIGCMIEIPSAALTVGSILKLVDFISVGTNDLIQYILSVDRGD 475

E.coli          468 DMISHLYQPMSPSVLNLIKQVIDASHAEGKWTGMCGELAGDERATLLLLGMGLDEFSMSA 527
C.freundii      468 DMISHLYQPMSPSVLTLIKQVIDASHAEGKWTGMCGELAGDERATLLLLGMGLDEFSMSA 527
K.pneumoniae    468 DMISHLYQPMSPSVLNLIKQVIDASHAEGKWTGMCGELAGDERATLLLLGMGLDEFSMSA 527
S.aureus        470 ERVSYLYQPYNPSILRLVKQVIEASHKEGKWTGMCGEMAGDETAIPLLLGLGLDEFSMSA 529
E.faecalis      472 ERVSYLYQPYNPSILRLIKNVIDAAHAEGKWAGMCGEMAGDQTAVPLLLGMGLDEFSMSA 531
N.gonorrhorae   476 DSVSHLYQPGHPSVLKMLQHVIRTANRMDKDVSVCGEMAGDTAFTRVLLGMGLRRFSMNP 535

E.coli          528 ISIPRIKKIIRNTNFEDAKVLAEQALAQ-PTTDELMTLVNKFIEEKTIC 575
C.freundii      528 ISIPRIKKIIRNTNFEDAKVLAEQALAQ-PTTDELMTLVNKFIEEKTIC 575
K.pneumoniae    528 ISIPRIKKIIRNTNFEDAKVLAEQALAQ-PTTDELMTLVNKFIEEKTIC 575
S.aureus        530 TSILKARRQINGLSKNEMTELANRAVDC-ATQEEVIELVNNYVK 572
E.faecalis      522 TSILKTRSLMKRLDTTKMAELADRALKECDTMEEVFALVEEY 573
N.gonorrhorae   536 NNILPVKNIILHSN 549
```

Figure S1. EI sequence alignment. The sequence of the *E. coli* EI is aligned against EI from other, randomly selected, drug-resistant bacteria: *Citrobacter freundii* (identity 97%; similarity

99%; gaps 0%), *Klebsiella pneumoniae* (identity 96%; Similarity 98%; gaps 0%), *Staphylococcus aureus* (identity 51%; similarity 70%; gaps 0%), *Enterococcus faecalis* (identity 49%; similarity 68%; gaps 1%), and *Neisseria gonorrhorae* (identity 36%; similarity 58%; gaps 2%). Residues forming the allosteric pocket are in red. Active site residues are in bold. Blue and red lines indicate the EIN and EIC domains, respectively.
